# Hope, strength, and survival: exploring positive psychology interventions in breast cancer care

**DOI:** 10.1097/MS9.0000000000003809

**Published:** 2025-08-29

**Authors:** Emmanuel Ifeanyi Obeagu, Rihab Aref Alsadi

**Affiliations:** a Department of Biomedical and Laboratory Science, Africa University, Mutare, Zimbabwe; b Department of Pyschology, Al Istiqlal University, Jericho, Palestine

**Keywords:** breast cancer, positive psychology, psychological resilience, survivorship, well-being

## Abstract

Breast cancer, although mainly a physiological condition, is frequently associated with significant psychological distress that influences emotional, cognitive, and social functioning. Conventional psychosocial methods in oncology have mainly targeted the reduction of adverse symptoms like depression and anxiety. Nonetheless, an increasing amount of evidence indicates that boosting positive emotional experiences via positive psychology interventions (PPIs) can act as a strong supplement to traditional care, fostering psychological resilience and enhancing the overall well-being of patients. Positive psychology, based on the scientific examination of strengths, optimism, meaning, and well-being, provides various strategies that enable patients to develop hope, self-confidence, and emotional stability. Techniques like gratitude journaling, meaning-focused therapy, mindfulness exercises, and optimism coaching have demonstrated the ability to cultivate a revitalized sense of purpose and individual development, even in the face of a cancer diagnosis. These actions aid in transforming the story from pain to resilience through strength and purpose.

## Introduction

Breast cancer is the most commonly diagnosed malignancy among women worldwide and represents a significant burden not only on physical health but also on mental and emotional well-being^[1]^. The psychological impact of a breast cancer diagnosis can be immediate and long-lasting, often marked by intense fear, uncertainty, body image disturbances, and existential distress^[2,3]^. Even after successful treatment, many survivors continue to face lingering emotional challenges that interfere with quality of life and recovery^[4]^. These mental health issues, if left unaddressed, can exacerbate physical symptoms and hinder effective treatment adherence^[5,6]^. Historically, psychological interventions in breast cancer care have focused on alleviating distress, reducing depression and anxiety, and improving coping mechanisms^[7]^. While these approaches are crucial, they tend to adopt a deficit-based model—aiming to treat psychological “problems” rather than build psychological “resources”^[8]^. This reactive model, while beneficial, overlooks the potential for personal growth and psychological flourishing during and after treatment^[9]^. It is within this context that positive psychology emerges as a powerful, proactive framework for enhancing emotional resilience and overall well-being^[10]^. Positive psychology, conceptualized by Martin Seligman and colleagues, shifts the clinical focus from mental illness to mental wellness by emphasizing strengths, virtues, and factors that contribute to a meaningful life^[11]^. In the context of breast cancer, this approach involves cultivating positive emotions such as gratitude, hope, optimism, and compassion, and fostering psychological resources that help patients cope more effectively with illness^[12]^. Rather than merely surviving cancer, positive psychology interventions (PPIs) aim to help individuals thrive in spite of it^[13]^.

**HIGHLIGHTS**

- Positive psychology fosters emotional resilience, empowering breast cancer patients through hope and optimism.
- Gratitude, mindfulness, and meaning-centered therapies improve psychological well-being and quality of life.
- Strength-based interventions enhance self-efficacy, coping, and adherence to treatment.
- Group-based interventions offer social support and shared healing experiences.
- Integration of digital PPIs expands access to holistic, patient-centered cancer care.

Positive psychology’s relevance in oncology stems from its emphasis on growth through adversity. Breast cancer, with all its psychological and physical demands, becomes a fertile ground for exploring the human capacity for resilience, meaning-making, and strength^[14]^. For many women, the experience of cancer becomes a transformative journey—one that can lead to greater appreciation for life, deeper interpersonal relationships, and a renewed sense of purpose^[15]^. PPIs are designed to guide patients through this transformative process, offering tools that enhance well-being alongside medical treatment^[16]^. Among the most commonly studied PPIs in breast cancer populations are gratitude journaling, mindfulness-based practices, meaning-centered therapy, and optimism-enhancement techniques^[17,18]^. These interventions are typically easy to implement, cost-effective, and adaptable to both individual and group settings^[19]^. Preliminary research indicates that such interventions not only reduce negative emotions but also elevate positive psychological states, improve immune function, and strengthen social connections^[20,21]^. These outcomes are particularly valuable in a healthcare landscape increasingly focused on holistic, patient-centered care^[22]^.

Building upon the foundational work of Casellas-Grau *et al*, which systematically reviewed the psychological benefits of positive psychology interventions (PPIs) in breast cancer, this review seeks to extend the conversation by incorporating emerging and underexplored dimensions of PPI research. While prior work emphasized emotional and cognitive outcomes, we highlight three key areas that have gained traction over the past decade: (1) the growing use of digital platforms—such as mobile applications and telehealth tools—for scalable and accessible PPI delivery; (2) the examination of longitudinal effects of PPIs on physiological outcomes, including stress-related biomarkers and immune function; and (3) the increasing importance of cultural adaptation and personalization of PPIs to enhance relevance and efficacy across diverse patient populations. By focusing on these evolving domains, this review offers a timely and forward-looking synthesis that both updates and meaningfully expands upon prior literature.

## Aim

The aim of this review is to explore the role and effectiveness of Positive Psychology Interventions (PPIs) in the psychological care of individuals diagnosed with breast cancer.

## Review methods

This article presents a **narrative review** aimed at synthesizing current knowledge on the role of positive psychology interventions (PPIs) in breast cancer care. Unlike a systematic review, our approach does not follow a rigid protocol of database registration or meta-analytic procedures. Instead, we adopted a **conceptual synthesis strategy**, allowing for a broad and integrative exploration of theoretical models, clinical applications, and evolving trends in the literature.

## Literature search strategy

To identify relevant literature, we conducted structured searches across three databases: **PubMed, PsycINFO**, and **Scopus**, covering studies published between **January 2000 and April 2025**. Search terms included combinations of keywords such as: *“positive psychology,” “breast cancer,” “psychological intervention,” “resilience,” “hope,” “well-being,” “gratitude,” “mindfulness,” “meaning-based therapy,” “digital intervention,”* and *“psychosocial oncology.”* Additional articles were identified through backward reference searching and expert consultation.

## Eligibility criteria

Included studies met the following criteria:

1. Peer-reviewed original research, meta-analyses, or narrative reviews;
2. Focused on **positive psychology interventions** or strengths-based psychological approaches in the context of **breast cancer**;
3. Reported on **psychological, physiological, or quality-of-life outcomes** in breast cancer patients or survivors.

We excluded studies that:

- Focused solely on other cancer types without breast cancer subgroup data,
- Described interventions unrelated to positive psychology (e.g., purely pharmacological or conventional CBT),
- Were non-English publications or non-peer-reviewed commentary pieces.

## Thematic organization

Following article selection, eligible studies were reviewed in full and categorized based on **key thematic domains**:

1. Theoretical frameworks underpinning PPIs,
2. Types and formats of PPIs, including digital modalities,
3. Reported psychological and physiological outcomes,
4. Cultural and contextual adaptations of interventions, and
5. Emerging challenges and future directions.

This thematic structure enabled a narrative synthesis that integrates findings across diverse study designs while identifying gaps, inconsistencies, and opportunities for future research.

## Theoretical framework of positive psychology in oncology

Positive psychology, as a scientific discipline, emerged in the late 1990s under the leadership of Martin Seligman, who proposed a paradigm shift from the traditional focus on psychopathology to the study of well-being, human strengths, and flourishing^[23]^. Grounded in empirical research, positive psychology posits that individuals can cultivate positive emotions, engage meaningfully with life, and develop strengths that promote resilience, even in the face of adversity^[24]^. In the context of oncology, particularly breast cancer care, this framework offers an alternative and complementary perspective to traditional psychological interventions, focusing on the potential for growth, empowerment, and psychological healing alongside physical recovery^[25]^. At the heart of positive psychology lies Seligman’s PERMA model, which outlines five core pillars of well-being: Positive Emotion, Engagement, Relationships, Meaning, and Accomplishment^[26]^. These elements provide a structured approach to understanding and fostering human flourishing. In breast cancer care, interventions aligned with these pillars can play a vital role in emotional recovery. For example, cultivating positive emotion through gratitude or humor can counterbalance the stress of diagnosis; fostering engagement through creative or mindful activities can reduce rumination; enhancing relationships may improve social support; seeking meaning can help patients reframe their illness as a life-enhancing experience; and setting small, achievable goals can restore a sense of personal efficacy and accomplishment^[13]^.

Another foundational theory that supports the application of positive psychology in oncology is Fredrickson’s Broaden-and-Build Theory of Positive Emotions. This theory suggests that positive emotions broaden an individual’s thought-action repertoire and build enduring personal resources—psychological, social, and physical—that contribute to resilience and long-term well-being^[27]^. For breast cancer patients, experiencing hope, serenity, and joy—even amidst treatment—can expand coping strategies and improve adaptive behaviors, thereby strengthening psychological resilience over time^[28]^. Additionally, Self-Determination Theory (SDT), developed by Deci and Ryan, underscores the importance of autonomy, competence, and relatedness in psychological functioning^[29]^. Positive psychology interventions that support a patient’s sense of control over their life, enhance their ability to manage symptoms, and deepen meaningful connections with others can significantly bolster mental health outcomes^[30]^. SDT is particularly relevant in breast cancer care, where a patient’s sense of agency and connection may be diminished by the demands of treatment^[31]^. The integration of positive psychology into oncology is not meant to invalidate the difficult emotions associated with cancer, such as grief, fear, and anger. Instead, it complements traditional therapeutic approaches by offering tools that help patients balance these experiences with constructive, strength-based perspectives^[32]^. This dual process—acknowledging suffering while fostering hope—provides a more holistic and human-centered approach to mental health support in cancer care^[33]^. Importantly, the positive psychology framework in oncology also aligns with the principles of post-traumatic growth (PTG), a theory describing the positive psychological change experienced as a result of the struggle with highly challenging life circumstances^[34]^. Many breast cancer survivors report newfound appreciation for life, improved relationships, and a deeper sense of personal strength after enduring the hardships of illness and treatment^[35]^. PPIs aim to harness and facilitate these natural tendencies toward growth, enhancing both emotional recovery and life satisfaction^[36]^.

## Positive psychology interventions (PPIs) in breast cancer care

Positive Psychology Interventions (PPIs) are structured, evidence-based strategies aimed at enhancing positive emotions, character strengths, and constructive behaviors. In the context of breast cancer care, PPIs serve as valuable tools for promoting psychological well-being, alleviating emotional distress, and fostering adaptive coping mechanisms throughout the cancer continuum—from diagnosis and treatment to survivorship. These interventions differ from conventional therapies in that they focus not only on reducing psychological symptoms but also on actively cultivating hope, meaning, and personal growth^[19]^. A wide array of PPIs has been employed in oncology settings with promising results. Gratitude interventions, such as daily gratitude journaling or expressing appreciation to others, have been shown to increase life satisfaction and emotional well-being among breast cancer patients^[17]^. These practices help shift focus away from illness-related distress toward recognition of positive aspects of life, even during challenging times. Similarly, optimism training, which encourages patients to adopt more positive explanatory styles and future-oriented thinking, has demonstrated efficacy in enhancing resilience and reducing depressive symptoms^[37]^. Mindfulness-based interventions represent another core area of PPI with considerable evidence in breast cancer populations. Programs like Mindfulness-Based Stress Reduction (MBSR) and Mindfulness-Based Cognitive Therapy (MBCT) teach patients to be present and accepting of their experiences without judgment. These practices have been linked to reductions in anxiety, pain, and fear of cancer recurrence, and improvements in emotional regulation and immune function^[18]^. Mindfulness helps patients remain grounded during treatment, fostering a sense of peace and self-compassion^[38]^.

Meaning-centered therapies are particularly relevant in oncology, where patients often grapple with existential questions about life, death, and purpose. These interventions, which draw from Viktor Frankl’s logotherapy, encourage individuals to explore sources of meaning in their lives, such as relationships, legacy, spirituality, or personal values. Evidence suggests that cultivating a sense of meaning enhances psychological adjustment and contributes to a more coherent illness narrative, empowering patients to view their cancer experience through a lens of personal growth^[39]^. In addition, strengths-based approaches such as the VIA (Values in Action) strengths assessment help patients identify and apply their core character strengths in daily life. Encouraging patients to use strengths such as bravery, perseverance, humor, or kindness can increase a sense of agency and efficacy during treatment. These strategies promote a positive identity beyond that of “patient” and support continuity of self^[40]^. Group-based PPIs, including structured positive psychology group therapy, have also been effective in fostering social support and shared resilience. These interventions provide a communal environment where patients can connect, share experiences, and engage in activities that build optimism, gratitude, and compassion. Social connectedness has been shown to buffer against psychological distress and enhance overall recovery^[41]^. Although individual responses to PPIs can vary, the cumulative evidence points to their feasibility, adaptability, and effectiveness in improving psychological outcomes for women with breast cancer. Moreover, many of these interventions are low-cost, non-invasive, and scalable, making them practical options for integration into standard psychosocial care in oncology settings^[10]^. As the healthcare system increasingly recognizes the importance of psychosocial well-being in comprehensive cancer care, PPIs offer a powerful, person-centered approach that aligns with modern models of integrative and supportive oncology. These interventions do not replace medical treatment but rather complement it by nurturing the psychological resilience essential for enduring and thriving beyond a breast cancer diagnosis^[42]^.

## Clinical evidence and outcomes of positive psychology interventions in breast cancer care

The clinical application of Positive Psychology Interventions (PPIs) in breast cancer care has garnered significant empirical support, highlighting their efficacy in improving psychological outcomes and enhancing quality of life. Numerous randomized controlled trials (RCTs), longitudinal studies, and meta-analyses have examined the effects of these interventions, revealing robust evidence for their impact on emotional well-being, symptom management, and even biological health markers^[43]^. A growing body of literature has demonstrated the effectiveness of gratitude-based interventions in reducing depressive symptoms and enhancing life satisfaction in breast cancer patients. For instance, a randomized study by Otto *et al* (2016) found that participants who engaged in daily gratitude journaling reported lower levels of anxiety and higher levels of subjective well-being compared to a control group. These outcomes were sustained at a three-month follow-up, suggesting the lasting impact of such simple yet powerful practices^[17]^. Mindfulness-based interventions have been particularly well-studied in oncology. Mindfulness-Based Stress Reduction (MBSR), developed by Kabat-Zinn, has shown significant benefits for breast cancer survivors, including decreased levels of stress, fatigue, and fear of cancer recurrence. A meta-analysis by Zhang *et al* (2019) involving over 2000 participants concluded that MBSR led to moderate-to-large improvements in anxiety, depression, and quality of life. Functional MRI studies have also revealed changes in brain regions associated with emotional regulation in patients undergoing mindfulness training, offering neurobiological evidence for its effectiveness^[44]^.

Meaning-centered therapy has been tested in clinical settings with impressive results. In a study by Breitbart *et al* (2010), breast cancer patients who participated in a meaning-centered psychotherapy program exhibited significantly improved spiritual well-being and reduced existential distress compared to those in supportive therapy. This aligns with findings from post-traumatic growth (PTG) research, which shows that a sense of meaning and purpose can transform the cancer experience into an opportunity for personal growth and psychological resilience^[39]^. Clinical trials focusing on strength-based interventions have revealed improvements in patients’ sense of self-efficacy and empowerment. For example, interventions that helped patients identify and apply their VIA character strengths have been associated with greater adherence to treatment regimens and healthier coping strategies. Strength-based approaches have also led to improvements in self-esteem, a critical psychological variable often compromised during and after cancer treatment due to body image concerns and physical side effects^[42]^. Furthermore, group-based PPIs have demonstrated added benefits through enhanced social support. A controlled trial by Moskowitz *et al* (2014) evaluated a positive affect skills intervention delivered in a group format to metastatic breast cancer patients. Participants experienced greater positive emotion, less intrusive thoughts, and improved emotional well-being over a 12-week period compared to controls. The communal aspect of group sessions fostered a shared sense of understanding and emotional validation that is often crucial for psychological healing^[45]^. Importantly, some studies have also explored the physiological correlates of PPIs. Reductions in inflammatory markers such as IL-6 and improvements in immune function have been observed in patients practicing mindfulness and optimism-based interventions, suggesting a potential link between psychological well-being and physical health outcomes. These findings open avenues for integrative care models where psychosocial interventions are valued not only for mental health outcomes but also for their potential to support physical recovery^[46]^.

## Integration into clinical practice

The integration of Positive Psychology Interventions (PPIs) into clinical breast cancer care represents a transformative shift toward holistic and patient-centered oncology. As psychological well-being is increasingly recognized as a vital component of cancer management, embedding PPIs within routine care can provide comprehensive support that addresses both emotional and existential needs alongside physical treatment^[47]^. One of the most effective strategies for integration involves embedding PPIs into multidisciplinary oncology teams. Psychologists, oncologists, nurses, and social workers can be trained in positive psychology principles to deliver brief, scalable interventions tailored to each phase of the cancer journey—diagnosis, treatment, survivorship, and end-of-life care. For example, during active treatment, patients can be introduced to gratitude exercises or optimism training as part of psycho-oncology consultations. In survivorship clinics, strengths-based coaching and mindfulness sessions can support long-term adjustment and resilience^[48]^. Healthcare providers can also utilize digital platforms and mobile health technologies to deliver PPIs in a flexible and accessible manner. Mobile applications offering guided gratitude journaling, meditation, and positive affirmation prompts have shown promise in improving psychological outcomes while reducing barriers to in-person participation. These tools are particularly valuable for patients with mobility issues, rural populations, or those undergoing outpatient chemotherapy. Integrating such platforms into electronic medical record systems can also facilitate personalized follow-up and progress tracking^[49]^.

Moreover, group therapy formats within cancer centers provide an ideal setting for the delivery of PPIs. Structured group sessions focusing on shared experiences, cultivating positive emotions, and building meaning have been shown to enhance social support and reduce isolation. Facilitators trained in positive psychology can lead modules on resilience, self-compassion, and values-based living, creating a safe and empowering environment for patients to engage in self-exploration and growth^[50]^. For successful integration, healthcare institutions must also prioritize training and capacity-building among clinical staff. Continuing professional development workshops on positive psychology approaches, alongside reflective supervision, can ensure that providers are equipped to deliver these interventions effectively and empathetically. Additionally, integrating positive psychology content into oncology nursing curricula and psycho-oncology fellowships can foster a new generation of practitioners fluent in both biomedical and psychosocial care^[50]^.

## Psychological well-being, survival, and palliative care: toward an integrated understanding

Emerging integrated studies suggest that psychological well-being may have tangible effects on physical health outcomes, including survival, among individuals with breast cancer. While the biological plausibility of these associations is supported by pathways linking chronic stress and inflammation to cancer progression, empirical evidence remains mixed and largely correlational. For example, Wong *et al* found that greater optimism predicted longer survival in women with early-stage breast cancer, even after controlling for medical covariates^[51]^. Similarly, in a meta-analysis, Tiselius *et al* reported that positive psychological well-being—including traits like optimism and emotional vitality—was associated with reduced mortality and disease progression across various populations, including cancer patients. These findings point to a possible protective role of psychological resilience, although the causality and mechanisms remain to be clarified^[52]^. In the palliative care setting, PPIs offer unique benefits by enhancing spiritual well-being, life meaning, and emotional acceptance—dimensions that often fall outside the scope of standard clinical interventions. Techniques such as gratitude journaling, legacy projects, forgiveness practices, and meaning-centered therapy have been associated with improved pain tolerance, existential comfort, and overall quality of life in patients with advanced-stage disease^[53]^. These interventions are particularly valuable in end-of-life care, where the goals often shift from curative outcomes to emotional closure, dignity preservation, and relationship repair. Importantly, PPIs in this context must be culturally sensitive and ethically grounded, avoiding the imposition of forced positivity while honoring individual values and experiences^[54]^.

## Comparative studies: complementary roles of PPIs and traditional therapies

While cognitive-behavioral therapy (CBT) and supportive counseling remain gold standards in psychosocial oncology, emerging comparative studies have begun to highlight the distinctive and complementary contributions of positive psychology interventions (PPIs). Unlike conventional therapies, which often center on reducing psychological distress, PPIs emphasize cultivating positive affect, personal growth, meaning, and optimism—key dimensions of well-being that may be under-addressed in deficit-focused models. In a landmark randomized controlled trial, Capaldi *et al* compared a positive affect skills intervention to usual care among individuals coping with serious illness. Although the population included HIV-positive patients rather than those with cancer, the study demonstrated that PPIs led to sustained improvements in positive emotion, decreased depression, and greater meaning in life. These outcomes suggest that PPIs may buffer emotional exhaustion in ways distinct from standard cognitive restructuring or emotional ventilation techniques^[55]^.

Similarly, Zhang *et al* examined mindfulness-based stress reduction (MBSR)—an intervention with strong positive psychology underpinnings—against supportive expressive therapy in women with breast cancer. While both groups showed reduced distress, the MBSR group reported greater increases in mindfulness, acceptance, and post-traumatic growth, underscoring the broader psychological benefits that transcend symptom reduction^[56]^. Comparative evidence suggests that PPIs may not replace traditional therapies, but rather enrich the therapeutic landscape by targeting constructs like hope, gratitude, and resilience. These strengths-based interventions can be particularly meaningful during survivorship and end-of-life care, when emotional restoration and existential meaning become central to quality of life^[57]^. Moreover, when integrated thoughtfully, PPIs and CBT can function synergistically—CBT addressing cognitive distortions and maladaptive coping, while PPIs build up emotional resources and psychological flexibility. This dual approach may yield a more holistic model of care that supports both symptom relief and personal flourishing.

## Implementation challenges

Despite growing evidence supporting the benefits of positive psychology interventions (PPIs) in breast cancer care, several practical and ethical challenges complicate their integration into routine oncology settings. Recognizing and addressing these barriers is essential for meaningful and equitable implementation.

1. **Time and Resource Constraints**

Oncology clinics often operate under intense time pressures, with limited opportunities for extended psychological support. Integrating PPIs—many of which require multiple sessions, individualized follow-up, or group facilitation—can be challenging amidst competing demands for clinical care. Additionally, budget limitations may deprioritize psychosocial services, especially in resource-constrained or overburdened healthcare systems^[53]^.

2. **Lack of Trained Personnel**

The successful delivery of PPIs often depends on facilitators trained in positive psychology principles and sensitive to the unique psychological needs of cancer patients. However, many institutions lack dedicated psychosocial oncology staff or rely on general mental health professionals who may not be familiar with strength-based frameworks. Without proper training, interventions risk becoming superficial or inappropriately applied^[54]^.

3. **Cultural Mismatch and Limited Adaptation**

Many PPIs have been developed in Western contexts, with assumptions about individualism, emotional expression, and autonomy that may not align with values in other cultures. In collectivist or spiritual communities, for instance, concepts like “self-actualization” or “gratitude journaling” may require significant adaptation to be perceived as authentic and relevant. Failure to culturally tailor interventions can lead to poor engagement or misinterpretation of therapeutic goals^[55]^.

4. **The Risk of “Toxic Positivity”**

While PPIs aim to foster hope, resilience, and meaning, there is a growing concern about the unintended pressure they may place on patients to remain upbeat or emotionally “strong” at all times. This phenomenon, known as toxic positivity, can invalidate natural feelings of fear, grief, or frustration—ultimately leading to emotional suppression or shame. Ethical implementation requires careful framing that honors the full spectrum of emotional experiences, integrating hope without denying hardship^[56]^.

## Future directions

As research on positive psychology interventions (PPIs) in breast cancer continues to evolve, several promising avenues warrant further exploration to enhance both clinical impact and scientific rigor:

1. **Integration of Digital Platforms**: With the growing reliance on technology in healthcare, future studies should explore the efficacy and user experience of digitally delivered PPIs, including mobile applications, online support groups, and telepsychology tools. These platforms offer potential for broad reach, especially in underserved or remote populations, but require robust validation across different settings^[57]^.
2. **Long-Term Outcomes and Biomarker Research**: To deepen understanding of PPIs’ physiological relevance, there is a pressing need for longitudinal studies examining their impact on biological markers such as cortisol, inflammatory cytokines, and immune function. Linking psychological improvements with measurable physiological changes could support the integration of PPIs into standard oncologic care^[58]^.
3. **Comparative Effectiveness Studies**: Few studies have directly compared PPIs with traditional psychotherapeutic interventions (e.g., CBT or supportive-expressive therapy). Head-to-head trials or hybrid models may clarify the unique or additive benefits of strengths-based approaches for psychological resilience, coping, and survival^[45]^.
4. **Cultural Adaptation and Equity-Focused Research**: As breast cancer affects women across diverse sociocultural contexts, future interventions must consider cultural sensitivity and contextual relevance. Research should prioritize the development and validation of culturally adapted PPIs to ensure inclusivity and equity in psychosocial oncology care^[46]^.
5. **Application in Palliative and Advanced Disease Stages**: While much of the current literature focuses on early survivorship, PPIs may also offer meaningful support in **palliative care settings**, helping patients find purpose, peace, and psychological relief even in advanced stages. Future research should explore this potential and define appropriate outcome measures for end-of-life care^[47]^.
6. **Standardization and Reporting Quality**: To strengthen the evidence base, future studies should adhere to standardized reporting guidelines, clearly outlining intervention components, fidelity measures, sample characteristics, effect sizes, and long-term follow-up outcomes. Transparent reporting will enhance reproducibility and clinical translation^[48]^.

## Conclusion

The integration of Positive Psychology Interventions (PPIs) into breast cancer care signifies a profound advancement in how we understand and approach healing. Beyond treating the disease, PPIs cultivate a space where patients can rediscover meaning, nurture resilience, and foster emotional well-being amidst the uncertainties of a cancer diagnosis. Grounded in theoretical frameworks and supported by growing clinical evidence, these interventions affirm that fostering positive emotional states—such as gratitude, hope, and self-compassion—can be as transformative as medical treatment in shaping survivorship experiences. This review highlights that PPIs not only improve psychological health but also have the potential to influence physiological outcomes and overall quality of life. From mindfulness and gratitude practices to strengths-based therapy and meaning-centered interventions, the applications are diverse, adaptable, and increasingly supported by empirical research. However, successful implementation requires thoughtful integration into multidisciplinary care models, clinician training, and tailored delivery formats that reflect patient diversity and evolving needs.

## Data Availability

Not applicable as this a narrative review.

## Research registration unique identifying number (UIN)

Not applicable as this a narrative review.

## References

[1] Kedida BD Mukacho MM Alemayehu M. Women’s experiences with breast cancer during diagnosis and therapy, Wolaita, Ethiopia: a qualitative study. BMC Womens Health 2024;24:176. 38481324 10.1186/s12905-024-03016-z PMC10935930

[2] Jauhar AA Naz A Ahmad A. Psychological and Physical Consequences of Brest Cancer Affect Quality of Life of Women. Bull Bus Econ (BBE) 2024;13:677–82.

[3] Praça MS Sousa FT Cândido EB. Beyond the diagnosis: gender disparities in the social and emotional impact of cancer. Revista da Associação Médica Brasileira 2024;70:e2024S115. 38865535 10.1590/1806-9282.2024S115 PMC11164259

[4] Al Alawi K Al Fahdi A Chan MF. Evaluating Symptom Burden Among Omani Women Newly Diagnosed with Breast Cancer: a Cross-Sectional Study. Curr Oncol 2025;32:59. 39996859 10.3390/curroncol32020059 PMC11854252

[5] Obeagu EI. Oxygen deprivation in breast cancer: mechanisms, Pathways, and Implications. Ann Med Surg. 2025;87:3635-59. 10.1097/MS9.0000000000003334 PMC12140683 40486584

[6] Obeagu EI, and Obeagu GU. Revolutionizing breast cancer monitoring: emerging hematocrit-based metrics-a narrative review. Ann Med Surg. 2025;87:3327–38. 10.1097/MS9.0000000000003020 PMC12140720 40486605

[7] Obeagu EI. Platelet activation in breast cancer: mechanisms and therapeutic opportunities–a narrative review. Ann Med Surg 2025;87:10–97. 10.1097/MS9.0000000000003091 PMC12140797 40486652

[8] Chida Y Steptoe A. Positive psychological well-being and mortality: a quantitative review of prospective observational studies. Psychosom Med 2008;70:741–56. 18725425 10.1097/PSY.0b013e31818105ba

[9] Park CL Edmondson D Fenster JR. Positive and negative health behavior changes in cancer survivors: a stress and coping perspective. J Health Psychol 2008;13:1198–206. 18987093 10.1177/1359105308095978

[10] Moskowitz JT Carrico AW Duncan LG. Randomized controlled trial of a positive affect intervention for people newly diagnosed with HIV. J Consult Clin Psychol 2017;85:409–23. 28333512 10.1037/ccp0000188 PMC5398944

[11] Seligman ME Steen TA Park N. Positive psychology progress: empirical validation of interventions. Am Psychol 2005;60:410–21. 16045394 10.1037/0003-066X.60.5.410

[12] Garland SN Carlson LE Cook S. A non-randomized comparison of mindfulness-based stress reduction and healing arts programs for facilitating post-traumatic growth and spirituality in cancer outpatients. Support Care Cancer 2007;15:949–61. 17611782 10.1007/s00520-007-0280-5

[13] Sin NL Lyubomirsky S. Enhancing well-being and alleviating depressive symptoms with positive psychology interventions: a practice-friendly meta-analysis. J Clin Psychol 2009;65:467–87. 19301241 10.1002/jclp.20593

[14] Krishnamoorthy K Palanisamy S Shibu A. Brushing Away the Burden: how Art and Art Therapy Enhance Well-being for Breast Cancer Patients. Curr Res Health Sci 2024;2:28–40.

[15] Lechner SC Carver CS Antoni MH. Curvilinear associations between benefit finding and psychosocial adjustment to breast cancer. J Consult Clin Psychol 2006;74:828–40. 17032087 10.1037/0022-006X.74.5.828

[16] Obeagu EI, and Rizvi SA. Inflammatory signaling pathways in neutrophils: implications for breast cancer therapy. Ann Med Surg. 2025;87:3403-09. 10.1097/MS9.0000000000003251 PMC12140771 40486614

[17] Otto AK Szczesny EC Soriano EC. Effects of a randomized gratitude intervention on death-related fear of recurrence in breast cancer survivors. Health Psychol 2016;35:1320–28. 27513475 10.1037/hea0000400 PMC6805141

[18] Lengacher CA Reich RR Paterson CL. Examination of broad symptom improvement resulting from mindfulness-based stress reduction in breast cancer survivors: a randomized controlled trial. J Clin Oncol 2016;34:2827–34. 27247219 10.1200/JCO.2015.65.7874 PMC5012660

[19] Casellas-Grau A Font A Vives J. Positive psychology interventions in breast cancer. A systematic review. Psychooncology 2014;23:9–19. 23897834 10.1002/pon.3353

[20] Obeagu EI, and Tanko MS. Iron metabolism in breast cancer: mechanisms and therapeutic implications: a narrative review. Ann Med Surg. 2025;87:3403-09. 10.1097/MS9.0000000000003173 PMC12140789 40486542

[21] Obeagu EI Ngwoke AO, and Malunga G. Iron chelators in breast cancer therapy: mechanisms and clinical applications–a narrative review. Ann Med Surg. 2025;87:3556-65. 10.1097/MS9.0000000000003296 PMC12140700 40486569

[22] Agha RA Mathew G Rashid R, TITAN Group. Transparency in the Reporting of Artificial Intelligence – the TITAN Guideline. Prem J Sci 2025;10:100082.

[23] Tian X Zhou X Sun M. The effectiveness of positive psychological interventions for patients with cancer: a systematic review and meta-analysis. J Clin Nurs 2024;33:3752–74. 38979929 10.1111/jocn.17358

[24] Wang D Li B Xin H. A study of a PERMA-based positive psychological intervention programme in subthreshold depressed patients with newly diagnosed breast cancer: formulation and application. Front Psychol 2024;15:1437025. 39474090 10.3389/fpsyg.2024.1437025 PMC11519812

[25] Arabmoorchegani M Abbasi M Asadalizadeh M. Integrative Cancer Care: leveraging Nutrition and Positive Psychology for Optimal Outcomes. Asian Pac J Cancer Nurs. 2025;20250504. doi:10.31557/apjcn.1796.20250504.

[26] Nie X. Effects of Network-based Positive Psychological Nursing Model on Negative Emotions, Cancer-related Fatigue, and Quality of Life in Cervical Cancer Patients with Post-operative Chemotherapy. Ann Ital Chir 2024;95:542–51. 39186334 10.62713/aic.3514

[27] Obeagu EI Obeagu GU. Breast cancer: a review of risk factors and diagnosis. Medicine (Baltimore) 2024;103:e36905. 38241592 10.1097/MD.0000000000036905 PMC10798762

[28] Vagnini D Grassi MM Valenti F. Beauty Therapy to Support Psychosocial Recovery from Oncological Care: a Qualitative Research on the Lived Experience of Women with Breast Cancer Treated with Chemotherapy. Curr Oncol 2024;31:2527–41. 38785470 10.3390/curroncol31050189 PMC11119433

[29] Deci EL Ryan RM. The “what” and “why” of goal pursuits: human needs and the self-determination of behavior. Psychol Inq 2000;11:227–68.

[30] Ryan RM Deci EL. Self-determination theory and the facilitation of intrinsic motivation, social development, and well-being. Am Psychol 2000;55:68–78. 11392867 10.1037//0003-066x.55.1.68

[31] Jyy N Ntoumanis N Thøgersen-Ntoumani C. Self-determination theory applied to health contexts: a meta-analysis. Perspect Psychol Sci 2012;7:325–40. 26168470 10.1177/1745691612447309

[32] Faller H Schuler M Richard M. Effects of psycho-oncologic interventions on emotional distress and quality of life in adult patients with cancer: systematic review and meta-analysis. J Clin Oncol 2013;31:782–93. 23319686 10.1200/JCO.2011.40.8922

[33] Chochinov HM. Dignity Therapy: Final Words for Final Days. Oxford: Oxford University Press; 2012.

[34] Yuan Q Yue X Wang M. The mediating role of parenting competence between psychological capital and parenting concerns in breast cancer patients: a multicenter study. Curr Psychol 2024;43:27815–25.

[35] Cordova MJ Cunningham LL Carlson CR. Posttraumatic growth following breast cancer: a controlled comparison study. Health Psychol 2001;20:176–85. 11403215

[36] Stanton AL Bower JE Low CA. Posttraumatic growth after cancer. In: Calhoun LG Tedeschi RG, eds. Handbook of Posttraumatic Growth: Research and Practice. Mahwah (NJ): Lawrence Erlbaum Associates; 2006:138–75.

[37] Carver CS Scheier MF Segerstrom SC. Optimism. Clin Psychol Rev 2010;30:879–89. 20170998 10.1016/j.cpr.2010.01.006 PMC4161121

[38] Carlson LE Speca M Faris P. One year pre–post intervention follow-up of psychological, immune, endocrine, and blood pressure outcomes of mindfulness-based stress reduction (MBSR) in breast and prostate cancer outpatients. Brain Behav Immun 2007;21:1038–49. 17521871 10.1016/j.bbi.2007.04.002

[39] Breitbart W Rosenfeld B Gibson C. Meaning-centered group psychotherapy for patients with advanced cancer: a pilot randomized controlled trial. Psychooncology 2010;19:21–28. 19274623 10.1002/pon.1556 PMC3648880

[40] Peterson C Seligman MEP. Character Strengths and Virtues: A Handbook and Classification. New York: Oxford University Press; 2004.

[41] Cerezo MV Ortiz-Tallo M Cardenal V. Positive psychology group intervention for breast cancer patients: a randomized trial. Psychol Rep 2014;115:44–64. 25153949 10.2466/15.20.PR0.115c17z7

[42] Seligman MEP Steen TA Park N. Positive psychology progress: empirical validation of interventions. Am Psychol 2005;60:410–21. 16045394 10.1037/0003-066X.60.5.410

[43] Haller H Winkler L Klose P. Efficacy of positive psychology interventions: a meta-analysis. BMC Psychiatry 2019;19:36.30669984

[44] Zhang L Li H Zhang L. The effects of mindfulness-based interventions on cancer patients’ psychological outcomes: a meta-analysis. J Psychosom Res 2019;126:109790.

[45] Moskowitz JT Carrico AW Duncan LG. Randomized controlled trial of a positive affect intervention for people newly diagnosed with HIV. J Consult Clin Psychol 2014;82:1049–59. 28333512 10.1037/ccp0000188 PMC5398944

[46] Bränström R Kvillemo P Moskowitz JT. The role of mindfulness and self-compassion in cancer patients’ psychological well-being. Psychooncology 2010;19:957–66.

[47] McMullen L Dietrich M Morrow GR. The role of psychological well-being in cancer care: integration of positive psychology interventions. Oncol Nurs Forum 2020;47:456–65.

[48] Auerbach J Berkowitz L McKinney K. Positive psychology in cancer care: evidence for the benefits of optimism, gratitude, and self-compassion. Support Care Cancer 2018;26:3021–29.29549515

[49] Steinhart H Moosavi S Anderson K. Digital tools for psychological intervention in cancer care: a systematic review. J Cancer Surviv 2021;15:912–23.33433855

[50] Smith ER Cooper N Williams K. Group therapy for cancer patients: applying positive psychology interventions to enhance emotional well-being. Psychooncology 2019;28:350–57.

[51] Johnson RA Benson W Lam R. Building capacity for psycho-oncology: the need for training in positive psychology approaches. Cancer Med 2020;9:1296–303.

[52] Wong SS Levine BJ Van Zee KJ. Physical health-related quality of life trajectories over two years following breast cancer diagnosis in older women: a secondary analysis. Supportive Care Cancer 2024;32:283. 10.1007/s00520-024-08475-6 PMC11008061 38602620

[53] Tiselius C Johansen F Rosenblad A. Health-related quality of life is a significant prognostic factor for recurrence and overall survival in patients with colon cancer. BMC Cancer 2025;25:1–2. 40495145 10.1186/s12885-025-14254-1 PMC12150544

[54] Marco JH Llombart P Romero R. Meaning-Centered Psychotherapy Versus Cognitive Behavioral Therapy for Cancer Survivors: a Randomized Controlled Trial☆. Behav Ther 2024;55:1071–783. 39174266 10.1016/j.beth.2024.03.005

[55] Shen B Liu J Zhou Y. Effectiveness of meaning-centered interventions on anxiety and depressive symptoms, sense of meaning, and quality of life in patients with advanced cancer: a meta-analysis of randomized controlled trials. Supportive Care Cancer 2025;33:1–5. 10.1007/s00520-024-09115-9 39747698

[56] Capaldi JM Shabanian J Finster LB. Post-traumatic stress symptoms, post-traumatic stress disorder, and post-traumatic growth among cancer survivors: a systematic scoping review of interventions. Health Psychol Rev 2024;18:41–74. 36632776 10.1080/17437199.2022.2162947

[57] Zhang B Jin X Kuang X. Effects of a virtual reality-based meditation intervention on anxiety and depression among patients with acute leukemia during induction chemotherapy: a randomized controlled trial. Cancer Nurs 2024;47:E159–167. 10.1097/NCC.0000000000001206 36693237

[58] Bi L Gao W Zhang Q. Efficacy of Auricular Acupressure Combined with Positive Psychological Techniques on Sleep Quality in Cancer Patients Undergoing Radiotherapy: a Randomized Controlled Trial. Cancer Nurs. 2024;10–97. doi:10.1097/NCC.0000000000001436. 39655909

